# Morbidity and health-related quality of life of patients accessing laparoscopic sleeve gastrectomy: a single-centre cross-sectional study in one province of Canada

**DOI:** 10.1186/s40608-017-0176-y

**Published:** 2017-12-11

**Authors:** Laurie K. Twells, Shannon Driscoll, Deborah M. Gregory, Kendra Lester, John M. Fardy, Dave Pace

**Affiliations:** 1 Faculty of Medicine, Memorial University, Medical Education Building, 300 Prince Philip Drive, St. John’s, NL A1B 3V6 Canada; 2 School of Pharmacy, Memorial University, Health Sciences Centre, 300 Prince Philip Drive Newfoundland and Labrador, St. John’s, A1B 3V6 Canada; 3 Eastern Health, Health Sciences Centre, 300 Prince Philip Drive, St. John’s, NL A1B 3V6 Canada

**Keywords:** Severe obesity, Laparoscopic sleeve gastrectomy, Health-related quality of life, HRQoL

## Abstract

**Background:**

In Canada, severe obesity (BMI ≥ 35 kg/m^2^) affects 5% or 1.2 million adults. Bariatric surgery is the only effective treatment for severe obesity, but the demand for publicly funded procedures is high and capacity limited. Little is known in Canada about the types of patients undergoing these procedures, especially laparoscopic sleeve gastrectomy (LSG). The study objective is to examine the socio-demographic profile, morbidity and HRQoL of patients accessing LSG in one Canadian province.

**Methods:**

Health status and HRQoL were examined in patients (*n* = 195) undergoing LSG. HRQoL was assessed using the EQ-5D-3L, SF-12v2 and the Impact of Weight on Quality of Life-lite questionnaire.

**Results:**

Mean age and BMI were 44 and 49 kg/m^2^ and most were women (82%). Pre-surgery, comorbidities were sleep apnea (65%), dyslipidemia (48%), hypertension (47%) and osteoarthritis (44%). Patients reported impaired HRQoL with 44–67% reporting problems in mobility, usual activities, pain and anxiety/depression. Physical health was impaired more than mental health. There were few socio-demographic differences between women and men, but significant differences in comorbid conditions such as sleep apnea, dyslipidemia, hypertension and gout exist (*p* < .05). Women reported fewer problems with self-care (9.5% vs. 25.0%, p < .05), and better overall health (VAS 61.5 vs. 52.0, p < .05) and General Health (39.3 vs. 32.9, p < .05), but greater impairment in self-esteem (27.3 vs. 44.1, *p* < .01) and sexual life (49.2 vs. 63.6, p < .05).

**Conclusions:**

Before LSG, patients reported significant morbidity and impaired HRQoL. Although baseline characteristics were similar between men and women, gender specific differences were observed in comorbid profile and HRQoL.

## Background

In Canada, severe obesity, measured as a body mass index or BMI ≥ 35 kg/m^2^) affects on average about 5% or over 1.2 million adults and is projected to increase to 6.4% by 2019. The rate of severe obesity is slightly higher in women (5.7%) compared to men (4.6%). [1] Significant and durable weight loss can be achieved with the use of bariatric surgery as a treatment for severe obesity. [2–4] It improves the health status and life expectancy of those affected. [5–7] The eligibility criteria for accessing bariatric surgery includes the following: 1. BMI ≥ 40 kg/m^2^ or a BMI between 35 and 39.9 kg/m^2^ with a comorbidity and 2. unsuccessful weight loss attempts [8]. North America studies indicate that women are more likely than men to access bariatric surgery [9, 10].

In Canada, the volume and provision of bariatric surgery has increased. In 2012/2013 almost 6000 procedures were performed compared to less than 1600 procedures in 2006–2007 and the number of hospitals performing bariatric procedures increased from 34 to 46 over the same time period [9]. There is a disconnect between the supply and demand of bariatric surgery resulting in long wait times for patients that average 5 years [11–15]. There are significant regional variations in the provision of surgical services in Canada and inequities in access to surgery based on geographical and socio-economic factors [13, 16, 17]. In Canada, the percentage of patients that meet eligibility criteria for bariatric surgery is estimated to be about 1% [18]. Consequently bariatric surgery is available to very few individuals who could potentially benefit from it. This is further heightened by the absence of any universally accepted and judicious approach to triaging patients for surgery [13, 14, 19]. Laparascopic sleeve gastrectomy (LSG) is the most frequently performed bariatric surgery option in Canada. In North America, the number of LSG’s increased by 244% between 2011 and 2013. Forty-three percent of all bariatric surgeries are now LSG’s [20].

One of the most important patient-reported reasons for wanting to undergo bariatric surgery is reduced quality of life. [21] Measures of well-being, functioning, and health under physical, social, and psychosocial domains comprise the concept Health-Related Quality of Life (HRQoL). [22, 23] It is important to examine HRQoL in pre-surgical patients in order to analyze changes after surgery and over time. A recent publication highlights the limited number of studies available for meta-analysis for a variety of reasons (e.g., selective reporting of HRQoL, use of non-validated tools and inconsistent reporting and presentation of results) [24].

Little is known in Canada [9, 18] or internationally [25] about the types of patients undergoing these procedures. Examining data on socio-demographics, sex distribution, pre-surgery BMI, comorbid profile and patient reported quality of life will help to inform healthcare providers, payers and health practitioners about the profile of patients that seek, are referred to and access bariatric surgery in Canada. This information is critical in order to: evaluate outcomes post-surgery; aggregate and compare data across centres; include data in national or international registries.

The current study objective is to investigate the morbidity and HRQoL of patients accessing LSG at a single-centre in one Canadian province. A second objective is to determine whether differences in HRQoL exist between male and female bariatric surgery candidates.

## Methods

The current study is a cross-sectional analysis of the HRQoL of patients undergoing bariatric surgery at different time points in one centre in one province of Canada. Assessment of patients undergoing LSG takes place pre-surgery and post-surgery at 1, 3, 6, 12, 18, 24 months and annually thereafter. The provincial Health Research Ethics Authority (#11.101) approved the study and subjects provided written informed consent to take part.

### Setting

In response to the increasing demand for a treatment for severe obesity, Eastern Health, one of four regional health authorities established a provincial multi-disciplinary bariatric surgery program to offer bariatric surgery to its residents of Newfoundland and Labrador (~500,000, of which approximately 8% is potentially eligible for surgery) [9]. Based on surgeon preference and expertise, LSG is most often (98%) performed at this centre. The surgical technique used in the current study has been previously described [26]. Since 2011, 417 LSGs have been performed. This study examines the socio-demographic profile, morbidity and HRQoL of 200 consecutive patients accessing bariatric surgery in this newly established surgical program.

### Measures of health-related quality of life

We evaluated HRQoL in patients before surgery and by sex using three validated tools: the Euroqol-5 Dimension-3 level (EQ-5D-3L) [27], the Short-Form-12 version 2 (SF-12v2) [28] and a weight specific tool, the Impact of Weight on Quality of Life-Lite (IWQOL-Lite) [29]. The EQ-5D-3L, is an indirect preference-based health survey that consists of a 5 dimension descriptive system assessing mobility, self-care, pain, usual activity and anxiety each of which can be rated at one of three levels (no problems, some problems or extreme problems). These combine to create 243 possible health states. The descriptive system is scored using a set of weights that represent the general population’s preferences and allows for the calculation of a single summary preference-based utility index or score. EQ-5D-3L utility scores range between full health of 1 (where the respondent has no problems on any dimension) to the lowest score of −0.59 (where the respondent reports that they are at the bottom level of each dimension) [27]. An overall health EQ-5D-3L visual analogue scale (VAS) is also calculated. This score ranges from 0 (worst imaginable health) to 100 (perfect health) and is presented as a mean and standard deviation. The SF-12v2 is a short version of the SF-36, a validated tool used to assess quality of life. It has been validated against the SF-36 for patients with and without obesity [30]. The SF-12v2 survey contains 12 questions that assess 8 domains: physical function, role physical, bodily pain, general health, vitality, social function, role emotional and mental health. These domains are used to calculate a physical component score (PCS) and a mental health component scores (MCS) [28]. The PCS and MCS scores follow a T distribution (mean 50, SD 10), normalized for the general United States (US) population. License specific software accounts for any missing data and generates a mean score for each domain and for both component scores [28]. The IWQOL-Lite, a shorter form of the original questionnaire, assesses the impact of weight on quality of life in individuals exploring treatments for weight loss. The IWQOL-Lite includes 31 statements that start with “Because of my weight…” with response options to each statement ranging from (1) “Never true” to (5) “Always true” that measure the impact of weight on 5 domains (i.e., physical function, self-esteem, sexual life, public distress and work life). A score is calculated for each domain for each patient that answers at least 50% of the questions in any given domain. A total score is calculated if patients have responded to at least 26 out of the 31 questions. Raw scores are converted into a T-score (0–100), with 100 representing best possible health. Mean and standard deviations are reported for each domain [29].

### Statistical analysis

Descriptive analyses were performed for continuous variables that included the calculation of mean, standard deviation, standard error, median, interquartile range, minimum and maximum, and range. For categorical variables, data are presented as n and %‘s. IBM SPSS version 22.0 [31] was used to analyze survey data from the EQ-5D-3L and the IWQOL-Lite surveys. The EQ-5D-3L index values were calculated using US time trade-off (TTO) data. As required, the SF-12v2 results were analyzed using licenser specific software (i.e., Health Outcomes Software version 4.5) [28]. Differences by sex in demographic and individual survey results were determined using t-tests for continuous variables and Pearson’s chi-squared test for categorical variables and, where appropriate, the Fisher exact test is reported. Statistical significance was set at *p* < .05.

## Results

In May 2011 when the bariatric surgery program commenced, 200 consecutive pre-surgical patients were recruited to the Newfoundland and Labrador Bariatric Surgery Study. One hundred ninety-five patients (98%) completed the baseline questionnaires. Pre-surgery socio-demographic characteristics and comorbidity profile are presented in Tables 1 and 2. Overall 82% of patients accessing bariatric surgery were women. The average age, weight and BMI were 44 ± 10 years (range 22–70 years), 135 ± 23.2 kg and 49 kg/m^2^ ± 6.7, respectively. A small number of eligible patients gained weight between the initial assessment/ acceptance for surgery and actual surgery (*n* = 10) resulting in a range of BMI values between 35.2–67.2 kg/m^2^. The majority of the sample had post-secondary or some post-secondary education (75%) and were in full or part-time employment (62%). The average number of comorbidities reported were 5 and 74% of the sample reported ≥4 comorbidities. Obstructive sleep apnea (OSA) (65%), dyslipidemia (48%), back pain (51%), hypertension (47%), osteoarthritis (44%), gastroesophageal reflux disease (GERD) (43%) and type 2 diabetes mellitus (T2DM) (42%) were reported most often.

**Table 1:** Baseline characteristics of total sample and by sex

	Total population *n* = 195		Women *n* = 159		Men *n* = 36		*p*-value
	Mean (SD)	Range	Mean (SD)	Range	Mean (SD)	Range	
Age (years)	44 (10)	22–70	44 (9)	22–70	46 (11)	29–66	*p* = 0.110
Weight (kg)	134.9 (23.2)	90.0–231.0	130.2 (19.8)	90.0–187.5	155.8 (25.9)	106.0–231.0	P < .001
BMI (kg/m^2^)	48.8 (6.7)	35.2–67.2	48.7 (6.8)	35.2–67.2	49.6 (6.5)	37.8–60.7	*p* = 0.428
Obese Class II	16 (8.2%)		13 (8.2%)		3 (8.3%)		*p* = 0.975
Obese Class III	179 (91.8%)		146 (91.8%)		33 (91.7%)		
	n	%	n	%	n	%	
Female	159	81.5%	143		52		
Smoking							
Never	81	47.6%	65	45.5%	16	59.3%	*p* = 0.188
Former	53	31.2%	45	31.5%	8	29.6%	*p* = 0.850
Current	36	21.2%	33	23.1%	3	10.7%	*p* = 0.142
Marital Status							
Married/Common Law	140	72.2%	109	69.0%	31	86.1%	*p* = 0.039
Divorced/Separated/Single/ Never married/Widow	54	27.8%	49	31.0%	5	13.9%	
Education							
None/Some High School or diploma	47	24.6%	39	25.0%	8	22.9%	*p* = 0.790
Some post-secondary/Post-Secondary	144	75.4%	117	75.0%	27	77.1%	
Annual Income							
< $15,000	8	4.1%	7	4.4%	1	2.8%	*p* = 0.877
$15,000 - $29,999	24	12.3%	21	13.2%	3	8.3%	
$30,000 - $49,999	45	23.1%	37	23.3%	8	22.2%	
$50,000 – $79,999	38	19.5%	30	18.9%	8	22.2%	
≥ $80,000	53	27.2%	41	25.8%	12	33.3%	
Not Answered	27	13.8%	23	14.5%	4	11.1%	
Employment Status							
Full-time/Part-time	120	61.9%	98	61.6%	22	62.9%	*p* = 0.893
Other	74	38.1%	61	38.4%	13	37.1%	
Race							
Caucasian	179	95.2%	146	95.4%	33	94.3%	*p* = 0.776
Other	9	4.8%	7	4.6%	2	5.7%	
Average # of comorbidities (SD)	5.4 (2.7)	0–16	5.2 (2.8)	0–16	6.3 (2.3)	2–11	
Number of Comorbidities							
< 4	49	26.1%	45	28.8%	4	12.5%	*p* = 0.055
≥ 4	139	73.9%	111	71.2%	28	87.5%

**Table 2:** Comorbidity profile of total sample and by sex

	Total *n* = 195	Women *n* = 159	Men *n* = 36	*p*-value
	*n* (%)	*n* (%)	*n* (%)	
Sleep Apnea	127 (65.1)	94 (59.1)	33 (91.7)	*p* < .001
On CPAP	89 (70.1)	61 (64.9)	28 (84.8)	
Back Pain (*n* = 188)	95 (50.5)	80 (51.3)	15 (46.9)	*p* = 0.650
Dyslipidemia (*n* = 186)	89 (47.8)	68 (43.9)	21 (67.7)	*p* = 0.015
Hypertension (*n* = 188)	89 (47.3)	67 (42.9)	22 (68.8)	*p* = 0.008
Osteoarthritis (n = 188)	83 (44.1)	68 (43.6)	15 (46.9)	*p* = 0.733
Type 2 Diabetes	82 (42.1)	64 (40.3)	18 (50.0)	*p* = 0.285
Gastroesophageal Reflux Disease (*n* = 189)	81 (43.1)	64 (41.0)	17 (53.1)	*p* = 0.155
Gallbladder Disease/Gallstones (*n* = 187)	75 (40.1)	68 (43.6)	7 (22.6)	*p* = 0.029
Asthma (n = 188)	42 (22.3)	32 (20.5)	10 (31.3)	*p* = 0.184
Polycystic Ovary Syndrome (n = 159)	32 (20.1)	32 (20.1)	n/a	
Yeast/Fungal Infection (n = 187)	26 (13.9)	24 (15.5)	2 (6.3)	*p* = 0.169
Hypothyroidism (n = 187)	21 (11.2)	19 (12.3)	2 (6.3)	*p* = 0.327
Gout (n = 188)	21 (11.2)	9 (5.8)	12 (37.5)	*p* = 0.000

According to the EQ-5D-3L, patients reported some or extreme problems in mobility (47.2%), usual activity (53.1%), pain/discomfort (67.0%) and anxiety/depression (44.1%) with few reporting problems in self-care (12.4%) (Table 3). The average EQ-5D index and VAS scores were 0.78 ± 0.01SE and 59.8 ± 18.7SD, respectively. (data not shown) According to the SF-12v2, the PCS and MCS were 36.4 and 47.8 respectively, with normative scores of 50(10) (Table 4). As the SF-12v2 provides normative data for the general US population, it is possible to calculate what percentage of the current surgical sample provided scores below, at or above the population norm (mean 50 ± 10). For the total surgical sample, the percentages of patients that scored below, at or above the population norm were 77%, 21% and 2% for PCS and 36%, 29% and 35% for MCS. According to the IWQOL-Lite questionnaire, an instrument developed to specifically assess weight-related quality of life where lower scores (0–100) indicate greater impairment, the domains most impacted by weight were self-esteem (30.4), physical function (41.9), public distress (44.1), sexual life (51.8) and work (61.0). The IWQOL-Lite total score was 43.2 ± 18.7 (Table 5).

**Table 3:** EQ-5D-3L scores for total sample and by sex

	Mobility *n*(%)			Self-Care* *n*(%)			Usual Activities *n*(%)			Pain/Discomfort *n*(%)			Anxiety/Depression *n*(%)		
	Total	Women	Men	Total	Women	Men	Total	Women	Men	Total	Women	Men	Total	Women	Men
No Problems	103 (52.8%)	89 (56.0%)	14 (38.9%)	170 (87.6%)	143 (90.5%)	27 (75.0%)	91 (46.9%)	78 (49.4%)	13 (36.1%)	64 33.0%)	49 (31.0%)	15 (41.7%)	109 (55.9%)	87 (54.7%)	22 (61.1%)
Some/ Extreme Problems	92 (47.2%)	70 (44.0%)	22 (61.1%)	24 (12.4%)	15 (9.5%)	9 (25.0%)	103 (53.1%)	80 (50.6%)	23 (63.9%)	130 (67.0%)	109 (69.0%)	21 (58.3%)	86 (44.1%)	72 (45.3%)	14 (38.9%)
p-value	0.064			0.021			0.150			0.220			0.485		
Total	195	159	36	194	158	36	194	158	36	194	158	36	195	159	36

*p < .05 between men and women. T-tests were performed comparing “no problems” to “problems” (some and extreme problems were combined) between men and women

**Table 4:** SF-12v2 scores for total sample and by sex

	Overall *n* = 195	Women *n* = 159	Men *n* = 36	*p*-value
Domain	Mean (SD)	Mean (SD)	Mean (SD)	
Physical Function	37.1 (10.4)	37.3 (10.3)	36.3 (10.8)	0.618
Role Physical	39.0 (10.7)	39.6 (10.4)	36.3 (12.0)	0.095
Bodily Pain	40.9 (12.7)	40.9 (12.3)	41.0 (14.7)	0.953
General Health*	38.2 (10.6)	39.3 (10.4)	32.9 (9.8)	0.001
Vitality	43.0 (9.3)	43.5 (9.3)	41.0 (9.3)	0.158
Social Functioning	42.0 (12.1)	42.1 (12.0)	41.9 (12.3)	0.927
Role Emotional Health	44.4 (12.1)	44.2 (11.9)	45.2 (13.2)	0.645
Mental Health	47.0 (10.0)	46.6 (10.0)	48.8 (9.9)	0.229
Physical Health Component Score (PCS)*	36.4 (10.6)	37.2 (10.3)	33.3 (11.3)	0.048
Mental Health Component Score (MCS)	47.8 (10.7)	47.5 (10.9)	49.1 (9.9)	0.416

*p < 0.05 between men and women

**Table 5:** IWQOL-Lite scores for total sample and by sex

	Overall		Women		Men		*p*-value
Domain	*n*	Mean(SD)	*n*	Mean(SD)	*n*	Mean(SD)	
Physical Function	194	41.9 (20.7)	158	42.2 (20.1)	36	40.7 (23.6)	0.705
Self Esteem**	194	30.4 (26.4)	158	27.3 (24.3)	36	44.1 (30.9)	0.004
Sexual Life*	181	51.8 (32.1)	148	49.2 (31.9)	33	63.6 (30.6)	0.019
Public Distress	193	44.1 (25.2)	157	42.5 (24.7)	36	51.5 (26.2)	0.051
Work	185	61.0 (24.7)	153	60.3 (25.3)	32	64.8 (21.6)	0.341
Total score*	193	43.2 (18.7)	157	42.0 (17.7)	36	48.7 (21.9)	0.049

*p < 0.05 between men and women. **p < .01 between men and women

### Socio-demographic characteristics by sex

There were very few differences in baseline characteristics pre-surgery (Table 1). Women had a significantly lower weight than men (130.2kgs vs 155.8kgs, *p* < .001), although BMI was not different. Women were less likely to be partnered (69.0% vs 86.1%, *p* < .05). Women and men were similar in terms of age, income, education, employment status, ethnicity and smoking behavior.

### Pre-surgical Comorbid conditions by sex

The comorbidity profiles comparing women and men were somewhat different (Table 2). Men most often presented for surgery with OSA, hypertension, dyslipidemia, GERD and T2DM, while women presented with OSA, back pain, dyslipidemia, osteoarthritis and gallbladder disease. There were some statistically significant gender differences; compared to women, men reported more OSA, dyslipidemia, hypertension, and gout, while women reported double the prevalence of gallbladder disease/gallstones compared to men pre-surgery.

### Health related quality of life by questionnaire and sex

Table 3 presents the results from the EQ-5D-3L. Fewer women reported problems with self-care compared to men (9.5% vs. 25%, *p* < 0.05) and while fewer women reported problems with mobility (44.0% vs. 61.1%) and usual activities (50.6% vs 63.9%) compared to men, these differences were not significant. In contrast, more women reported problems with pain/ discomfort (69.0% vs 58.3%) and anxiety/depression (45.3% vs 38.9%) compared to men, although not significantly different. Women reported significantly higher scores (i.e., better self reported health) on the VAS (61.5 ± 18.5SD vs 52.0 ± 17.6SD, *p* = 0.009) compared to men, although there was no significant difference between the two on the EQ Index (0.79 ± .013SE vs 0.75 ± .031SE) which describes the patient’s health state [data not shown].

According to the SF-12v2 (Table 4), women reported significantly less impairment in General Health (39.3 vs 32.9, *p* < .05) and in the PCS (37.2 vs 33.3, *p* = 0.048). Based on the normative data for women, the percentage of the study sample whose PCS was below, at or above population norms correspond to: 75% below, 22% at, and 3% above the norm. The percentage of the study sample whose MCS was below, at or above the population norm was: 37% below, 27% at, and 36% above the population norm. For men, the PCS values corresponded to 83%, 11% and 6% below, at or above the norm and for the MCS, 31%, 39% and 31% reported scores below, at or above the norm [data not shown].

The IWQOL-Lite scores are presented in Table 5. Women reported significantly lower scores than men for self-esteem (27.3 vs. 44.1, *p* < 0.05) and sexual life (49.2 vs. 63.6, p < 0.05) suggesting that in women, these domains were more impaired by their weight. Men and women did not differ in scores for three of five domains (i.e., physical function, public distress or work). The IWQOL-Lite total score was significantly lower for woman compared to men (42.0 vs 48.7, p < 0.05).

## Discussion

Patients in this study averaged 44 years of age and had an average BMI of 49 kg/m^2^. The majority of the sample were: women with post-secondary or some post-secondary education in full or part- time employment. Health status was significantly impaired pre-surgery with patients reporting on average 5.4 comorbidities. OSA affected two thirds (65%) of the study sample while half of the sample reported having been diagnosed with hypertension (47%), dyslipidemia (48%) and back pain (51%). Almost half of the study sample reported having osteoarthritis (44%), T2DM (42%), GERD (43%) and gallbladder disease (40%). Pre-operatively, women and men did not differ significantly on select socio-demographics variables (i.e., age, income, education or employment status, although there were differences in comorbid profiles. A high prevalence of OSA was diagnosed in both sexes (65%), and rates were one third higher for men. Men also reported higher rates of dyslipidemia, hypertension and gout. In contrast, women reported double the rate of gallbladder disease/gallstones.

Pre-surgery HRQoL was significantly impaired in the study sample. Few patients reported problems with self-care; however, between 44% and 67% of patients reported problems with anxiety/depression, mobility, usual activities and pain and discomfort. Patient perceptions of general health and physical health were impaired more than mental health.

According to the weight-specific IWQOL-Lite scale, patients reported impairment on all scales (most to least impairment): Self Esteem, Physical Function, Public Distress, Sexual Life and Work. Significant differences in HRQoL were reported by women and men on self esteem, sexual life and the IWQOL total score.

Women self-reported better General Health as evidenced by higher scores on the SF-12v2 (39.3 vs. 32.9) and the VAS (61.5 vs. 52.0) and better Physical Health (PCS 37.2 vs. 33.3). Although 10% of the total patient sample reported problems with self care on the EQ-5D-3L, women reported significantly fewer problems in this area than men (9.5% vs. 25.0%). Based on the IWQOL-Lite, women reported greater weight associated impairment than men on Self Esteem (27.3 vs. 44.1) and Sexual Life (49.2 vs. 63.6).

Our study population, the majority of whom were women, is similar to other patients seeking treatment for severe obesity [2, 18, 32–34]. Patients undergoing bariatric surgery are more often female with an average pre-surgery weight and BMI of 124.5kgs and 46 kg/m^2^, respectively [2]. With respect to socio-demographics, our sample represents a high level of socio-economic status, with over 75% having some/full post-secondary education and half in higher income brackets. Findings on the relationship between socio-economic status and access to bariatric surgery are inconsistent, although in North America it appears that access to bariatric surgery has been reserved for those of higher social standing [18, 32, 35, 36]. This finding has been supported by publications that highlight inequities in access to bariatric surgery in Canada [13, 18]. Women and men had similar baseline characteristics, with the exception that women were less likely to be in partnered relationships, a finding similar to other studies [18, 32, 33, 37].

A very high level of obesity-related comorbidity was observed in the current study sample although the comorbid profile of patients seeking obesity treatment can vary signficantly by centre [18, 33, 38, 39]. In the Alberta population-based prospective evaluation of the quality of life outcomes and economic impact of bariatric surgery (APPLES) study, Padwal et al., assessed 150 bariatric surgical patients in Alberta, Canada. Compared to the current study, the APPLES authors reported higher rates of hypertension (61% vs. 47%) and dyslipidemia (60% vs. 48%) in their pre-surgery population, but similar rates of T2DM (42% vs. 44%) [38]. In contrast, compared to national Canadian data published on bariatric surgery recipients, obesity-related comorbidity was much higher in our study population with higher rates of hypertension (47% vs.13%), dyslipidemia (48% vs. 2.4%) and T2DM (42% vs. 21%) [18]. This variation may be partly explained by the fact that Newfoundland and Labrador has the highest rates of T2DM and CVD in Canada [40, 41]. Although, there may be potential under-coding of pre-existing conditions in administrative datasets when compared to prospectively collected data [18]. High levels of comorbidity may be one factor that motivates patients to seek treatment for severe obesity. [39, 42] In previous research conducted at our centre, individuals seeking treatment for severe obesity reported health concerns as the primary reason for wanting to lose weight, similar to other studies [21]. In the current study, the prevalence of comorbid conditions differed between women and men seeking treatment (e.g., OSA, dyslipidemia, hypertension, gout, and gallbladder disease/gallstones) similar to a study of over 200 patients undergoing bariatric surgery conducted in Germany from the University Hospital Heidelberg. In this study the authors examined patient expectations of surgery and collected data on baseline comorbidity. A similar prevalence of hypertension was reported with men more likely than women to be affected. Although much lower prevalences of OSA and dyslipidemia were reported, men were twice as likely to report being affected than women. [39] Gender differences in the rates of OSA and risk factors for CVD are often reported, but differences in the rates of other conditions are more inconsistent [33, 39, 43, 44].

Consistent with the results of other published studies, individuals seeking obesity treatment in the current study, demonstrated significantly impaired HRQoL compared to: the general population, individuals with overweight/obesity, or those with severe obesity not seeking surgical treatment. [23, 45–47] Studies consistently demonstrate that in individuals seeking treatment for severe obesity, physical health is impacted more than mental health [5, 32, 46, 48], although the magnitude of the impact varies. In the current study, the PCS of 36.4 is lower than the PCS of 41.5 reported by Warkentin et al., in the Canadian APPLES study [49] although the MCS score of 47.8 is more comparable to the APPLES results of 46.9. Compared to the Utah Obesity Study [50], a prospective cohort study of over 400 patients accessing Roux-en-Y Gastric Bypass (RYGB), the current study’s pre-surgery HRQoL scores are higher than those reported by the authors (PCS 31.4, MCS 41.4). In a study on pre-surgical patients conducted in Germany, the authors reported a similar PCS to the current study (34.3 versus 36.4), although the MCS was lower (42.1 versus 47.8). In both these studies the direction of the impairment remained similar in that physicial health was impacted more than mental health. [48] The EQ index of 0.78 reported in the current study is similar to that reported by the APPLES Study authors (0.79), and significantly lower than population norms (0.82). Surgical patients in the current study reported overall health (VAS 59.8) that was slightly lower than the APPLES study authors (VAS 63.6) and significantly lower than population norms (VAS 78.8) [49, 51].

Similar to other studies, patients seeking surgical treatment for severe obesity report significantly impaired weight-related HRQoL, although the level of impairment varies [23, 46, 50]. In the current study, the total IWQOL-Lite score (0–100), was 43.2, which was lower than scores reported by Padwal et al., in the APPLES study (IWQOL-Lite: 49.9) [47] or Belle et al., in the US Longitudinal Assessment of Bariatric Surgery (LABS), (IWQOL-Lite 46.8) [52] but higher than that reported by the Utah Obesity Study (IWQOL-Lite 41.8) [50]. These total scores are well below those reported by the general population (IWQOL-Lite 94.7) and lower than those reported by individuals living with severe obesity not seeking surgical treatment (IWQOL-Lite 54.9) [52]. Similar to other studies using North American data, in the current study, the domains most impacted by weight in decreasing order of impact were self-esteem, physical health, public distress, sexual life and work [37, 52], but are in contrast to data from Europe where patients report that weight impairs physical function more than self-esteem. [37, 46] It is an interesting finding that work life was reported as least impaired by weight in the current study and in other published studies [37, 52] as research suggests that absenteeism from work and more importantly presenteeism are much higher in individuals living with severe obesity compared to other BMI categories [53]. This is an area that could warrant further exploration.

### Gender analysis

Significant differences were found between women and men with respect to HRQoL. Women reported better General Health on the SF-12v2 (39.3 vs. 32.9) and the EQ-5D VAS (61.5 vs. 52.0) and better Physical Health (SF-12v2: PCS 37.2 vs. 33.3). This finding is inconsistent with studies by Kolotkin et al. who found that General Health was more impaired in women than men [33] and Karlsson [6] and Belle [52] who found no gender differences in General Health or physical HRQoL. In the current study, this may be partly explained by the fnding that fewer women reported having problems with Self Care compared to men (9.5% vs. 25.0%). Accordng to the IWQOL-Lite, weight impacted self-esteem and sexual life more in women than men.

Previous research on gender differences using the IWQOL-Lite has been inconsistent, however the weight-related impairment consistently affects women more than men in the domains self-esteem and sexual life [30, 33, 37, 52, 54, 55]. Stout et al., found no gender differences on the IWQOL-Lite scores. [54] In contrast, other studies have reported differences, but not in the same domains. Kolotkin et al. [33] and Belle et al. [52] report gender differences in self-esteem, sexual life, work and total score while White et al. [55] report gender differences in physical function, self-esteem, sexual life and total score. In a study by Caxias et al., [37] assessing HRQoL in bariatric surgery treatment seeking individuals in North America, gender differences are limited to self-esteem, sexual life and total score with women reporting greater impairment than men. The consistency of study findings in this area and across numerous studies suggest that weight negatively impacts these psychosocial domains in women more than men and may be a leading reason for why women seek treatment for severe obesity four to five times more often than men in North America. Previous qualitative research has shown that women seeking bariatric surgery are more likely than men to have weight and body image concerns. [56] The gender difference in uptake of bariatric surgery may also be partly explained by the fact that women seek out health care services in general and for mental health concerns more often than men [57].

Strengths of this study include: the use of three validated instruments to assess generic and obesity-specific HRQoL; the availability of key socio-demographic variables and comorbidity data to allows for exploration of and gender comparisons among these variables. Examining gender differences in HRQoL may provide a potential explanation for this reported imbalance of bariatric surgery seeking behaviors of women as opposed to men. This study also has some limitations. First, it is difficult to infer a causal relationship exists between severe obesity and HRQoL in a cross-sectional study. Methodological challenges of reverse causality and temporality are inherent in this study design. Second, most comorbidities were self-reported. Third, we did not have access to specific data on depression, although the EQ-5D-3L data reports that almost half the study sample reported problems with depression/anxiety, and there were no gender differences observed. Although depression is often seen as an important determinant of HRQoL, studies report varying degrees of depression in patients seeking bariatric surgery contingent on assessment type (i.e., self-report versus diagnosis by a healthcare professional) that range from one to two thirds of the population, with inconsistent results on gender differences. [33, 49, 52] Finally, as the current study took place in one Canadian province, it may lack generalizability to other populations; however, the socio-demographics, comorbid profile and HRQoL of our patients are comparable to those in North America and other centres [9, 32, 52].

Future research should explore reasons why men are less likely to seek out bariatric surgery than women as this may signal potential sex-related disparities in access to bariatric surgery [35, 36]. For example, there may be inherent biases in referral patterns for bariatric surgery or different health-seeking behaviors between women and men. [57] A better understanding of why weight appears to impact women more than men in psychosocial areas is also warranted especially in the context of societal pressures [13, 33, 56]. In addition, the impact of surgery on HRQoL in the short, medium and specifically long term should be explored. The publication of HRQoL data 2 to 4 years after bariatric surgery for this study sample is planned.

## Conclusion

Our study results demonstrate that similar to other programs in North America and elsewhere, patients living with severe obesity who access bariatric surgery tend to be women with high socio-economic status. Individuals seeking treatment report significant morbidity and impaired HRQoL. Women and men presented with substantial but significantly different pre-operative comorbid profiles. HRQoL was significantly impaired in men and women. Women compared to men reported better scores in general and physical health and fewer problems with self-care. However, weight impaired women’s sexual life and self-esteem significantly more than men.
